# Energy transfer in *N*-component nanosystems enhanced by pulse-driven vibronic many-body entanglement

**DOI:** 10.1038/s41598-023-46256-z

**Published:** 2023-11-15

**Authors:** Fernando J. Gómez-Ruiz, Oscar L. Acevedo, Ferney J. Rodríguez, Luis Quiroga, Neil F. Johnson

**Affiliations:** 1 Departamento de Física Teórica, Atómica y Óptica, Universidad de Valladolid, 47011 Valladolid, Spain; 2 Instituto de Física Fundamental IFF-CSIC, Calle Serrano 113b, 28006 Madrid, Spain; 3 Escuela de Ciencias Básicas, Institución Universitaria Politécnico Grancolombiano, Bogotá, D.C 110231 Colombia; 4 Departamento de Física, Universidad de los Andes, A.A. 4976, Bogotá, D.C Colombia; 5 Physics Department, George Washington University, Washington, D.C 20052 USA

**Keywords:** Materials science, Biomaterials, Condensed-matter physics, Materials for devices, Materials for energy and catalysis, Materials for optics, Nanoscale materials, Theory and computation

## Abstract

The processing of energy by transfer and redistribution, plays a key role in the evolution of dynamical systems. At the ultrasmall and ultrafast scale of nanosystems, quantum coherence could in principle also play a role and has been reported in many pulse-driven nanosystems (e.g. quantum dots and even the microscopic Light-Harvesting Complex II (LHC-II) aggregate). Typical theoretical analyses cannot easily be scaled to describe these general *N*-component nanosystems; they do not treat the pulse dynamically; and they approximate memory effects. Here our aim is to shed light on what new physics might arise beyond these approximations. We adopt a purposely minimal model such that the time-dependence of the pulse is included explicitly in the Hamiltonian. This simple model generates complex dynamics: specifically, pulses of intermediate duration generate highly entangled vibronic (i.e. electronic-vibrational) states that spread multiple excitons – and hence energy – maximally within the system. Subsequent pulses can then act on such entangled states to efficiently channel subsequent energy capture. The underlying pulse-generated vibronic entanglement increases in strength and robustness as *N* increases.

## Introduction

There has been an exciting development over the past decade concerning chemical, biophysical and physical systems driven by short bursts of energy (e.g. laser) in which the subsequent energy processing involves coherence phenomena^1–6^. Certain aspects of this have been reported as arising from quantum-mechanical interference interactions between the electronic and vibrational constituents within the system. Examples of such systems include photosynthetic complexes^7–19^, metal surfaces^20–22^, molecular magnets^23–25^, biochemical control^26–30^, organic devices^31–33^, and more general nanostructures^34,35^. Understanding the impact of a strong, time-dependent perturbation (e.g. pulse) on many-body quantum mechanical correlations, is a necessary step in fully understanding how energy is processed in time in such systems – and its consequences for energy transfer. However, typical theoretical analyses of such systems cannot easily be scaled to the large numbers *N* of components in either a naturally occurring real sample or an artificially made device, and tend to average over or truncate memory effects.

The present paper is motivated by this outstanding theoretical challenge of treating the dynamics of the pulse on the same footing as the dynamics of the general *N* many-body quantum nanostructure system. The literature on such many-body quantum systems contains various theoretical methods which have been extended to include complications such as non-Markovianity, other forms of perturbations, and quantum phenomena like coherence and entanglement. Particularly notable examples include the reduced hierarchical equations of motion theory^36,37^, path-integral Monte Carlo^38,39^, the quasi-adiabatic path-integral algorithm^40,41^, and the time-dependent Davydov ansatz^42^. These methods typically entail a significant computational overhead and their complicated details may obscure the interplay between various competing physics effects. For a comprehensive review of these numerical methods, we refer to Ref.^36,42^.

There is an important caveat to our aims and hence this paper: we are not aiming at a detailed understanding of a specific real-world system with highly complex chemistry such as the light-harvesting complex LHC-II, but instead our focus is on advancing the basic physics understanding of time-dependent response in a many-body quantum system driven by a pulse of arbitrary strength. Since the problem is impossible to solve exactly for arbitrarily strong pulses in a system with complex chemistry such as LHC-II, we adopt the well-known approach of physicists of focusing entirely on a minimal model. While such a model is effectively a ‘toy’ for theoreticians like us to play with, this approach is well-established in Physics where seemingly simple models like the Ising model are employed to mimic collective behavior in systems with exceedingly complex chemical properties. Specifically, we include the time-dependent pulse as part of a so-called Dicke-like Hamiltonian of *N* two-level systems – not as a perturbation – and then we solve this model’s time-dependence exactly. Though this sacrifices most of the chemical details which could ultimately prove to be important for a specific real-world system (e.g. LHC-II), we show that doing so has the advantage of making the model exactly solvable numerically for any *N* without making any assumptions about the pulse being less than a certain strength, and without making any approximations concerning memory effects in the system’s quantum mechanical evolution. Hence our purposely minimal model simplifies all the complexities of any real-world nanostructure (e.g. chemistry) in order to explore the physical interplay of the dynamics of the pulse and the dynamics of the many-body quantum system with any $N>1$. We make no claims that this model is an accurate description of a complex system such as LHC-II – however our minimal model’s exact numerical solution does reveal new physical collective behavior that should transcend such chemical details and hence could arise in such systems, i.e. we show for the first time that pulses of intermediate duration generate highly entangled vibronic (i.e. electronic-vibrational) states that spread multiple excitons – and hence energy – maximally within the system. Subsequent pulses can then act on such entangled states to efficiently channel subsequent energy capture. The underlying pulse-generated vibronic entanglement increases in strength and robustness as *N* increases. The possibility of such effects in a complex system such as LHC-II at very short timescales, cannot therefore be ruled out.

**Figure 1:**
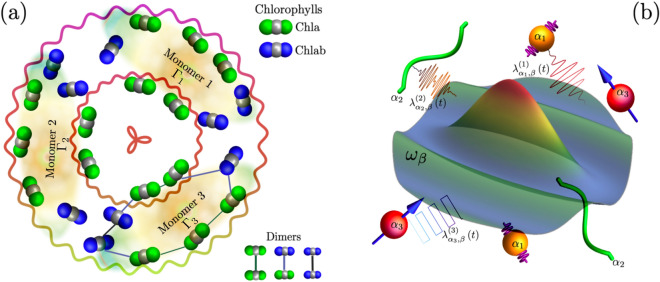
Potential applications. **(a)** Schematic of the light-harvesting LHC-II system containing various large-scale so-called ‘monomers’, each of which is an aggregate containing chlorophyll dimers^5,43^. We refer to Refs.^5,43^ for full chemical details. Zooming in, each chlorophyll molecule comprises a central magnesium atom (gray) and two nitrogen atoms^43^. The nitrogen atoms for Chla (Chlb) are colored green (blue). These chlorophylls are densely packed within a confined space of approximately $\sim 50$ Å. Associated with each of these three ‘monomers’, there are pairs of chlorophylls that can potentially form dimers and that form approximate two-level systems with very similar energy splittings^5^: this suggests that LHC-II contains $N=3$ chlorophyll dimer two-level systems with similar energy splittings akin to our minimal model^5^. Specifically, when stripped of all its chemical details, the system is a candidate $N=3$ system, i.e. three chlorophyll dimers with similar energy splittings can hence act approximately as $N=3$ two-level systems. **(b)** Schematic of a more general nanostructure system represented by our minimal model. The overall complex system comprises various types of nanostructure components denoted by $\alpha _i$, immersed within a bosonic bath. Each mode within this bath possesses an elemental frequency $\omega _\beta$. Each nanostructure component $\alpha _i$ can be effectively approximated as a two-level system. Furthermore, each nanostructure component is subject to the influence of a time-dependent pulse represented by $\lambda _{{\alpha _i},\beta }^{(i)}$ (see Eq. 1). Figures created by the authors using Mathematica version 13 https://www.wolfram.com/mathematica/ and also Microsoft Powerpoint version 16 https://www.microsoft.com/en-us/microsoft-365..

With this important caveat in mind, we will proceed in this paper to explore this minimal theoretical physics model and we will show that it has interesting new physics outcomes when solved exactly numerically. We simply speculate that it may serve as a very simplified model of complex systems such as LHC-II and hence its novel properties and results reveal previously overlooked behaviors in such systems. We will therefore refer to LHC-II in what follows and present it in our figures, in order to explain its fascinating structure to a broader audience and also to act as an illustrative example in order to motivate the general problem. We also hope that doing this will motivate an eventual full-scale calculation, using future computing power, in which all the chemistry details of prior models can somehow be included on the same footing as the time-dependent pulse, so that the full system can be solved exactly as here. Such light-harvesting complexes (LHC-II) are found in green plants (width $\sim 50$ Å) and play a pivotal role in capturing solar energy. Figure 1a shows a schematic of the LHC-II structure, adapted from Ref.^43^: it consists of an overall trimeric arrangement housing 24 chlorophylls and it is composed by two nitrogen and one magnesium atoms. These chlorophylls are organized into two irregular circular rings, as illustrated in Fig. 1a. The inner ring, situated at the core of the trimer, comprises six Chla molecules believed to play a crucial role in energy transfer. The remaining chlorophylls are strategically arranged to facilitate efficient absorption of incident light energy from all directions (for more details see Ref.^43–45^). Figure 1b gives a schematic representation of general nanostructure systems represented by our minimal model (Eq. 1) for which LHC-II may be an example. Adopting the simplest scenario, we approximate each individual nanostructure component as a two-level system. The two states $\left| X_{i} \right\rangle$ and $\left| Y_{i} \right\rangle$, represent the higher and lower energy states for the *i*-th nanostructure component (i.e. for the *i*-th dimer in the case of LHC-II where each dimer is a chlorophyll pair^5^). In LHC-II which features $N=3$ chlorophyll-pair dimers with similar energy splittings, the wavefunction for each dimer’s higher excitonic state $|X_\Gamma \rangle$ is more localized on the site with higher energy, while the lower state $|Y_\Gamma \rangle$ is more localized on the site with lower energy, hence a transition $|X_\Gamma \rangle \rightarrow |Y_\Gamma \rangle$ represents excitonic energy transfer in space from one to the other. This system motivates the focus in this paper on $N=3$, though our main findings hold for more general *N* (Fig. 1b).

Summarizing the rest of the paper, we will proceed to set up and solve numerically the real-time evolution of our minimal model. We will then show that pulses of intermediate duration generate strong *N*-body vibronic entanglements (i.e. between the electronic and vibrational subsystems) which represent a novel quantum-mechanical form of coherence, and which enhance the transfer and subsequent channeling of energy across the system. Our calculations predict that the strength and robustness of these *N*-body vibronic entanglements, increase with *N*. Despite our minimal model’s simplicity, it therefore yields novel results that are the first to our knowledge to account for the full many-body entanglement evolution for $N\ge 3$, specifically, the full pulse dynamics and full memory effects. In this way, it avoids the typical previous approximations of small *N*, linear response and truncated memory effects. Again we stress that our intention is not to address the longer timescale mechanisms driving natural photosynthesis in hot, wet environments^46^. Instead, our results help deepen understanding of the temporal quantum evolution in pulsed systems of general size *N* and we speculate that this can shed new light on the early-time kinetics in such real-world open systems, since in this temporal regime the timescale is too short to couple in many of the complex degrees of freedom that will naturally exist in such real-world systems^47^.

## Our minimal model

As is well known from classical and quantum optics, any incident electromagnetic (light) field ${\vec {E}}(t)$ generates an internal polarization field ${\vec {P}}({\vec {r}},t)$ within the material, given exactly by Maxwell’s Equations^48,49^. Though nonlinear and anisotropic in general, the presence of $\partial ^2/{\partial t^2}$ terms for both means that a pulse in ${\vec {E}}$ will generate a similar pulse in ${\vec {P}}$, and hence a pulse in the internal electric field dynamics coupling the electronic and vibrational systems^48,49^. Since we are not focusing on single-photon phenomena, a similar conclusion follows from a quantum-mechanical starting point^50,51^. This helps motivate our minimal model which is a Dicke-like Hamiltonian featuring time-dependent electronic-vibrational coupling $\lambda (t)$ (throughout the manuscript, we employ $\hslash =1$):

1 $$\begin{aligned} {H_N}(t)=\sum _\beta \omega _\beta a_\beta ^{\dagger }{a_\beta } + \sum _{i=1}^{N}\sum _{\alpha _i\in i}\frac{\varepsilon _{\alpha _i}}{2}{\sigma }_{z,\alpha _i}^{i} +\sum _\beta \sum _{i=1}^{N}\sum _{\alpha _i\in i} \frac{\lambda _{\alpha _i,\beta }^{(i)}(t)}{\sqrt{N}}\left( a_\beta ^{\dagger }+{a_\beta }\right) {\sigma }_{x,\alpha _i}^{i} \end{aligned}$$

where *N* is the number of components that respond to the pulse. We then integrate numerically this Hamiltonian system’s time-dependent equation in order to obtain its time-dependent quantum mechanical solutions. Other degrees of freedom that remain inert on such short timescales, can be neglected in this initial study but could be included later on through perturbation theory. ${\sigma }_{p,\alpha _i}^{i}$ denotes the two-level Pauli operators for excitation $\alpha _i$ on each component *i* with $p=x,z$. Here, we consider the elemental excitation energy for each nanostructure component $\alpha _i$ denoted by $\epsilon _{\alpha _i}$. The vibrational modes $\beta$ may or may not be localized, and can include relative modes^16^. In this context, the operator $a_{\beta }^{\dagger }$ ($a_{\beta }$) is responsible for generating (eliminating) a photon within the mode $\beta$ with energy $\omega _\beta$. $\lambda _{\alpha _i,\beta }^{i}$ corresponds to an individual time-dependent pulse applied to the nanostructure component $\alpha _i$ within mode $\beta$. Generally, each pulse can assume a unique time-dependent form tailored for experimental control purposes. Though Eq. (1) obviously sacrifices the chemical details of any given real-world system, it focuses attention on the key physical factors controlling the system’s complex quantum evolution. In terms of the speculated application to LHC-II, experimental observations of LHC-II suggest that the dimers exhibit a close resonance between their two excitonic levels and one of the background vibrational modes. Specifically, within the Chlb$_{601}$-Chla$_{602}$ dimers, the hybridized exciton energy-level splitting stands at $\epsilon =667.7$ $\hbox {cm}^{-1}$, while a vibrational mode with $\omega _{\textrm{vib}}=742.0$ $\hbox {cm}^{-1}$ is present^5^. Under the resonant conditions considered here, an $\epsilon =\omega =1$ pair is retained. As we show in this paper, this means in principle that many-body states can now be generated that entangle the electronic and vibrational subsystems as a result of the speed of changes in $\lambda (t)$ but *without* having to access the strong-coupling regime^50,52^. Many variants of Eq. (1) will show similar results due to an established universal dynamical scaling^52^ and the fact that they typically generate similar types of phase diagrams and hence have similar collective states in the static $\lambda$ limit^53–56^. Full background to our numerical calculations, including the multiple checks that we performed for convergence, are given in Refs. ^57–62^.

## Results and discussion

### Transfer of energy

In order to simulate a driving pulse, we consider a time-dependent coupling term in Eq. 1, $\lambda (t)$, with an up-down linear ramping in which the coupling strength goes from $0\rightarrow 1$ and then symmetrically $1 \rightarrow 0$. The pulse is characterized by a specific ramping velocity $\upsilon$, where $\upsilon ^{-1}$ sets the pulse duration. Hence, the driving pulse is expressed as follows:

2 $$\begin{aligned} \lambda \left( t \right) ={\left\{ \begin{array}{ll} \upsilon t,&{} 0\le t \le \upsilon ^{-1}, \\ 2-\upsilon t,&{} \upsilon ^{-1} < t \le 2\upsilon ^{-1}. \end{array}\right. } \end{aligned}$$

The particular choice of $\lambda$ given by Eq. (2), implies a round-trip protocol. Recently, the round-trip protocols has garnered interest in the context of quantum scaling properties in critical systems^63,64^. We numerically solve the time-dependent Schrödinger equation to obtain the instantaneous system state $\left| \psi \left( t \right) \right\rangle$.

We now examine in more depth the illustrative scenario where a single type of component is present $\alpha _i = \alpha$ and there are three of them ($N=3$), inspired by LHC-II with its three chlorophyll dimers of similar energies. We will focus on initially separated matter-vibrational states with zero vibrational excitations. Within each two-level system, there are two hybridized excitonic states $|X_\Gamma \rangle$ (higher excitonic energy state) and $|Y_\Gamma \rangle$ (lower excitonic energy state). For a simple asymmetric double-well system, akin to the chlorophyll-pair dimers in LHC-II, the wavefunction for the higher dimer state $|X_\Gamma \rangle$ is more localized on the site with higher energy, and the lower dimer state $|Y_\Gamma \rangle$ is more localized on the site with lower energy. Hence a transition $|X_\Gamma \rangle \rightarrow |Y_\Gamma \rangle$ represents a spatial transfer of exciton energy in space from one to another^5^. Importantly for light-harvesting, the $|Y_\Gamma \rangle$ wavefunction is localized on the chlorophyll *nearest* to an energy exit site. Hence any transition of exciton(s) from state(s) $\{|X_\Gamma \rangle \}$ to $\{|Y_\Gamma \rangle \}$ represents a spatial transfer of their energy toward a site where that energy can be easily exported. The initial states of the entire system including the vibrational modes are as follows: $\left| \psi _1\left( 0 \right) \right\rangle =\left| 1_g \right\rangle \otimes \left| 0 \right\rangle$ and $\left| \psi _2\left( 0 \right) \right\rangle =\left| W_2 \right\rangle \otimes \left| 0 \right\rangle$, respectively. The multi-excitonic states $\left| 1_g \right\rangle$, $\left| W_1 \right\rangle$, and $\left| W_2 \right\rangle$ can be expressed in the conventional Dicke-like manifold basis $\left| J,J_z \right\rangle$ as follows: $\left| 1_g \right\rangle =\left| 3/2,-3/2 \right\rangle$, $\left| W_1 \right\rangle = \left| 3/2,-1/2 \right\rangle$, and $\left| W_2 \right\rangle = \left| 3/2,1/2 \right\rangle$, where $J=3/2$ and $J_z$ takes on the values $-3/2$, $-1/2$, and 1/2. These states can also be expressed in the excitonic basis of the $N=3$ two-level systems as follows, indicating a total of 0, 1 and 2 excitons respectively:

3a $$\begin{aligned} \left| 1_g \right\rangle&\equiv \left| Y_1,Y_2,Y_3 \right\rangle , \end{aligned}$$

3b $$\begin{aligned} \left| W_1 \right\rangle&\equiv \frac{1}{\sqrt{3}}\left( \left| Y_1,Y_2,X_3 \right\rangle +\left| Y_1,X_2,Y_3 \right\rangle +\left| X_1,Y_2,Y_3 \right\rangle \right) , \end{aligned}$$

3c $$\begin{aligned} \left| W_2 \right\rangle&\equiv \frac{1}{\sqrt{3}}\left( \left| X_1,X_2,Y_3 \right\rangle + \left| X_1,Y_2,X_3 \right\rangle + \left| Y_1,X_2,X_3 \right\rangle \right) . \end{aligned}$$

For example in the state $\left| W_2 \right\rangle$, two matter excitations are shared among the three nanostructure components. We then evaluate the time-dependent probability of finding the system’s instantaneous state projected onto a given reference quantum state, pure $\left| \phi _f \right\rangle$ or mixed $\rho _f$ states. In general, the fidelity or probability for the instantaneous system’s density matrix $\rho \left( t \right) =\left| \psi \left( t \right) \right\rangle \left\langle \psi \left( t \right) \right|$ is given by

4 $$\begin{aligned} {P\left( t \right) :=\left[ \textrm{Tr}\sqrt{\sqrt{\rho _f}\rho \left( t \right) \sqrt{\rho _f}} \right] ^{2}.} \end{aligned}$$

**Figure 2:**
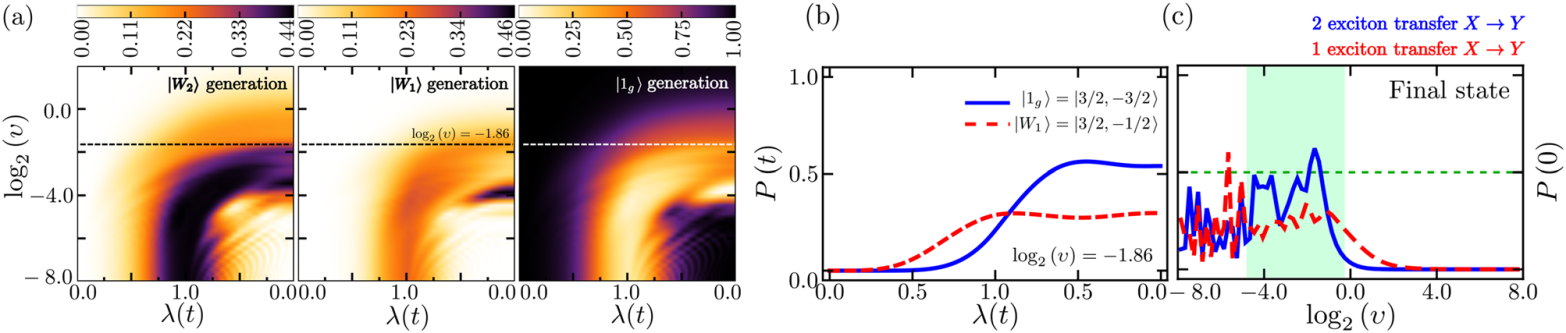
Example of $N=3$. **(a)** Colors show time-dependent probability profile of generating particular $N=3$ body multi-exciton states (left, middle and right panels), as a function of time during a single up-down pulse (horizontal axis) while the vertical axis shows the logarithm of the ramping velocity $\upsilon$. In all cases the matter initial state is $\left| 1_g \right\rangle$. The panels **(b)** and **(c)** show the contrast of impact when the starting state is $\left| 1_g \right\rangle$ or $\left| W \right\rangle _2$. **(b)** Time-dependent probability of multi-exciton transfer from $\left| X_\Gamma \right\rangle$ to $\left| Y_\Gamma \right\rangle$ (i.e. to lower energy chromophore in LHC-II) during pulse of intermediate duration. **(c)** Final state probabilities following pulse, as function of inverse pulse duration $\upsilon$. At intermediate durations (shown as green shaded in **(c)** and explicitly in **(b)**) the transfer and hence energy transport of 2 excitons has higher final probability than that of 1 exciton. Figures created by the authors using Mathematica version 13 https://www.wolfram.com/mathematica/ and also Microsoft Powerpoint version 16 https://www.microsoft.com/en-us/microsoft-365.

Information about the electronic (matter) subsystem is extracted from the corresponding reduced matrix, $\rho _{e}\left( t \right) =\textrm{Tr}_{v}\left[ \rho \left( t \right) \right]$ where $\textrm{Tr}_{v}[{\ldots }]$ means that the vibrational states have been traced out. In Fig. 2a, we depict the time-dependent probability profile (fidelity) given by direct evaluation of Eq. 4 starting with the total state given by $\left| \psi _1\left( 0 \right) \right\rangle =\left| 1_g \right\rangle \otimes \left| 0 \right\rangle$, where the state $\left| 0 \right\rangle$ correspond to zero excitations in the bosonic (i.e. vibrational) mode. In each panel, the corresponding reference state is shown in the upper part of the frame. In Fig. 2b, we have considered a scenario in which the complex system is initially prepared in the state $\left| \psi _2 (0) \right\rangle =\left| W_2 \right\rangle \otimes \left| 0 \right\rangle$. We have computed the probabilities of the matter subsystem reaching the ground state $\left| 1_g \right\rangle$ (represented by the solid blue line) and the state $\left| W_1 \right\rangle$ (indicated by the dashed red line) for a specific velocity $\upsilon$. In contrast, Fig. 2c displays the probabilities at the end of the pulse, with the matter subsystem initially prepared in the ground state.

**Figure 3:**
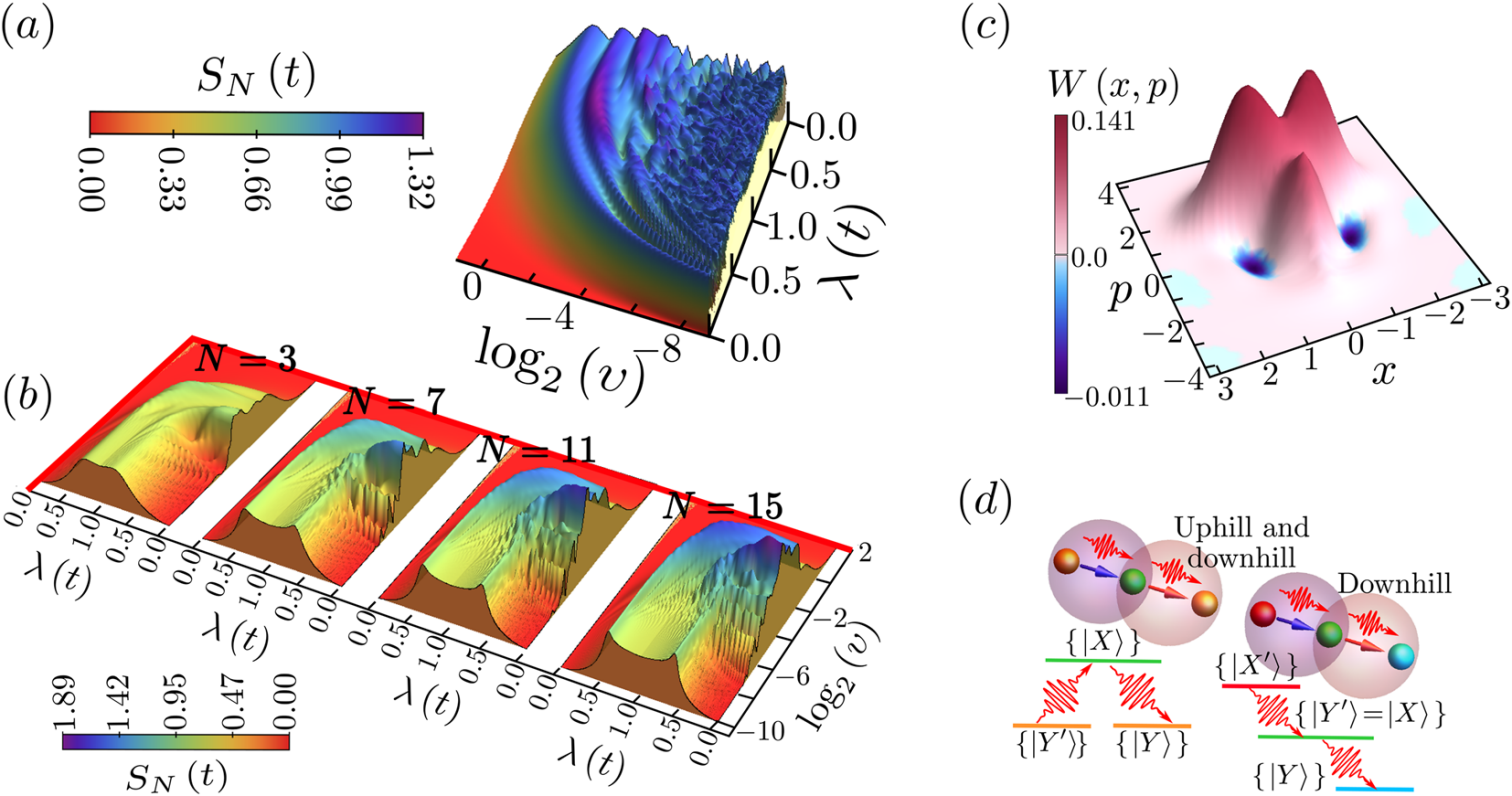
**(a)** Vibronic (i.e. electronic-vibrational) entanglement, as measured by the von Neumann entropy $S_N$, for $N=3$ system during an up-down pulse $\lambda (t)$, starting in the higher probability state $W_2$ as in Fig. 2b. Shown as a function of the logarithm of the inverse pulse duration (i.e. ramping velocity) $\upsilon$. **(b)** Vibronic (i.e. electronic-vibrational) entanglement $S_N$ for increasing *N*, starting in the initial $t=0$ ground state (i.e. $|\frac{3}{2},-\frac{3}{2}\rangle$ for $N=3$). In **(a)** and **(b)**, the vibronic entanglement (i.e. quantum coherence) is largest where shading is darkest. **(c)** Vibrational subsystem Wigner function ($N=3$) for the final state after the up-down pulse, $\lambda (t)$, at intermediate $\log _2[{\upsilon }] = -1.86$, confirming the non-classicality of the vibrational system’s post-pulse state. **(d)** Schematic of possible energy transport across the system (Fig. 1a): during each step, the excitonic energy is interchanged with quantized vibrations, the total wavefunction is time-evolving, mutual entanglement between electronic and vibrational quanta develops, but the full system’s state remains pure. Figures created by the authors using Mathematica version 13 https://www.wolfram.com/mathematica/ and also Microsoft Powerpoint version 16 https://www.microsoft.com/en-us/microsoft-365.

### *N*-body entanglement

In Figs. 2, 3, we now show how pulses in the electronic-vibrational coupling $\lambda (t)$ enhance the generation, spreading, and eventual channeling of multi-exciton states across an *N*-component system (e.g. Fig. 1) and that the cause lies in the *N*-body vibronic entanglement that the pulse generates and manipulates in time (i.e. time-dependent quantum mechanical coherence between the electronic and vibrational subsystems, Fig. 3b). Figure 2a considers initial state given by Eq. (3a), which is the electronic part of the $t=0$ ‘ground’ eigenstate of Eq. (1) ($\lambda (0)=0$) comprising the lower excitonic states $\{|Y_\Gamma \rangle \}$ in any three near-resonant nanostructure components: for example, the three near-resonant dimers in Fig. 1a. Additionally, after a single up-down pulse of moderate duration (i.e. intermediate $\upsilon$), the final state is dominated by $W_2$-states in which two excitations are shared among the three higher energy nanostructure components, and the entanglement spreads these maximally (Fig. 2a for $W_2$).

This means that if quantum coherence within one of the three nanostructure components is lost, the state of the remaining two nanostructure components remains entangled. This dominant $W_2$ generation is due to the high vibronic entanglement (i.e. many-body quantum coherence as measured by the von Neumann entropy $S_N$, Fig. 3a, b) that is generated between the excitonic and vibrational subsystems by intermediate duration pulses $\lambda (t)$. For the electronic subsystem, the von Neumann entropy is defined as follows:

5 $$\begin{aligned} S_N (t)=-\textrm{tr}\left\{ \rho _{e}\left( t \right) \log \left[ \rho _e \left( t \right) \right] \right\} ,\quad \text {where}\;\rho _e\left( t \right) =\textrm{tr}_v \left\{ \left| \psi \left( t \right) \right\rangle \left\langle \psi \left( t \right) \right| \right\} \end{aligned}$$

In this context, we consider a bipartition where subsystem *v* corresponds to the vibrational mode, and subsystem *e* encompasses the molecular excitonic component, within a total pure state $\left| \psi \left( t \right) \right\rangle$. In such a pure state, the entropy of subsystem *e* is inherently equal to the entropy of its complementary subsystem *v*. This quantity, denoted as $S_N$, serves as a measure of the entanglement existing between these two subsystems.

Since we are dealing with a closed system, characterized by a pure global quantum state that undergoes unitary evolution, any increase in $S_N$ within each subsystem signifies an exchange of information between the vibrational and matter constituents during the entire cycle. This observation lends itself to a more direct thermodynamic interpretation of the memory effects observed throughout the cycle. Figure 3b shows that the strength of this many-body quantum coherence and the range of pulse durations (i.e. inverse $\upsilon$) over which it exists, both *increase* with *N*. Unlike Fig. 2a, the probability of generating two excited exciton states in three independent nanostructure components would be lower and the state would not be entangled. Our calculations therefore predict that an intermediate duration pulse $\lambda (t)$ will generate *N*-body vibronic entanglement that efficiently spreads multiple excited excitons within the system, and that this effect will become even stronger as *N* increases.

Figures 2b, c and 3a illustrate the influence of a pulse $\lambda (t)$ on a system initially in the $W_2$ state, as generated previously. Notably, with a relatively high probability (approximately $\sim 0.5$), the application of a moderate-duration pulse, characterized by intermediate $\upsilon$, facilitates the transfer of both excitons across one nanostructure component to another. This transfer culminates in the state $|Y_1,Y_2,Y_3\rangle$. This process of transferring excitations can be illustrated using a simple model depicting the movement and transport of excitons: a single dimer ($N=1$) starting in state $|X\rangle$, would produce a final state $|Y\rangle$ with probability $\sim 0.5$, in line with simple arguments based on Rabi oscillations in a two-level system and Ref.^5^: hence for three independent ($N=1$) dimers in the same two-excitation initial state (e.g. $|X_1\rangle |X_2\rangle |Y_3\rangle$), the corresponding probability of ending in $|Y_1\rangle |Y_2\rangle |Y_3\rangle$ is $\sim (0.5)^3=0.125$. Hence for the LHC-II, this would mean that the pulse $\lambda (t)$ has manipulated the *N*-body vibronic entanglement in order to more efficiently transfer the excitons, and hence energy, to chlorophylls closest to the exit points across the trimer (i.e. to $\{|Y_\Gamma \rangle \}$).

Irrespective of whether biophysical systems naturally use these features or not, these features could be exploited in future nanostructure device designs for energy and information processing^52,57, 58, 65–69^ – in particular, using structures such as LHC-II given their natural abundance^43–45^. This suggests that by changing the choice of dimer in Fig. 1a, and hence the definition of lower and upper state and thus on which chromophores the excitations primarily lie, multiple highly-entangled excitations can be generated by individual pulses, transferred quantum mechanically between chromophore pairs across the trimer, and can hence reach the chromophores closest to exit sites, e.g. ‘uphill and downhill’ $\{|Z\rangle \}\rightarrow \{|X\rangle \}\rightarrow \{|Y\rangle \}$ (Fig. 3d), and ‘downhill’ $\{|X'\rangle \}\rightarrow \{|Y'\rangle \equiv |X\rangle \}\rightarrow \{|Y\rangle \}$, or any combination of these. This would occur without the need for energy relaxation processes to drive the direction of energy flow, hence the total wavefunction remains in a pure many-body quantum state – and it does not require strong electronic-vibrational coupling.

Though we take $\lambda (t)$ to be a piecewise linear up-down ramping of duration $\upsilon ^{-1}$ (i.e. the inverse ramping velocity), similar results will occur for any other up-down functional form since the strong electronic-vibrational (i.e. vibronic) entanglement at intermediate $\upsilon$ has its roots in path interference caused by two crossings of the quantum critical point $\lambda _c=0.5$^57–59^. Hence the details of the path are relatively unimportant. In particular, the shaded forms in Figs. 2a, 3a, b can be understood by averaging over the quantum oscillations generated by the up-down path interference in a simple two-level Landau-Zener-Stuckelberg picture: the average probability that the system ends up in the excited state manifold is $P^e=2P(1-P)$, where $P=\textrm{exp}(-2\pi \Delta ^2/4\upsilon )$ and $\Delta$ is the minimum effective two-level energy gap during the pulse. Approximating $\Delta \sim \lambda _c=0.5$, this predicts that $P^e$ increases monotonically as $\upsilon$ increases from the adiabatic regime, before falling off as $\textrm{log}_2 (\upsilon )\rightarrow 0$, exactly as seen in Figs. 2a, 3a, b.

Another method to analyze the indicators of non-classical behavior within the vibronic subsystem involves the Wigner quasi-probability distribution. The overall state of the mode is succinctly represented through its Wigner quasi-probability distribution^70^,

6 $$\begin{aligned} W\left( z,\rho _v \right) =\sum _{m=0}^{\infty }\left( -1 \right) ^{m}\left\langle m \right| {\mathscr {D}}^{\dagger }\left( z \right) \rho _v{\mathscr {D}}\left( z \right) \left| m \right\rangle , \end{aligned}$$

In this context, the conventional displacement operator is defined as ${\mathscr {D}}(z) = \exp \left[ z a^{\dagger } - z^*a\right]$, where $a^{\dagger }$ (*a*) represents the creation (annihilation) operator of the vibrionic mode, and *z* is a complex number. Figures 3a–c confirm the intrinsic non-classical nature of these processes generated by the pulse $\lambda (t)$. Figure 3b indicates that as *N* increases, the final states should be dominated by increasingly higher entangled equivalents of *W* states that entangle successively higher numbers of excited exciton states ($\{|X\rangle \}$) which can then be manipulated by subsequent pulses to efficiently channel energy toward $\{|Y\rangle \}$ states and hence energy exit points. Given the naturally-occurring availability of $N>3$ aggregates^43–45^, this enhancement with increasing *N* may inspire new device designs. The lower bound $\upsilon _{\textrm{min}}$ in Fig. 3b does not depend on the maximum value of $\lambda (t)$ reached. Instead, the scaling $\upsilon _{\textrm{min}} \propto N^{-1}$ comes from a relation for the minimal energy gap at the critical threshold^58^. The upper bound $\upsilon _{\textrm{max}}$ does not depend on system size, and is instead dictated by the $\textrm{log}_2 (v)\rightarrow 0$ drop-off of $P^e$. Figure 3c illustrates the purely quantum features that are present in the Wigner functions of the vibrational and electronic subsystems (e.g. negative values).

Though our results consider ramping up to the modest value of $\lambda (t)\approx 1$ and back, similar results occur for smaller maximum values and hence weaker pulses as long as maximum $\lambda (t)\ge 0.5$. For maximum $\lambda (t)< 0.5$, the system does not feel the quantum critical point and hence there is negligible entanglement, in line with empirical observations that quantum coherence effects are primarily found beyond the weak perturbative driving field regime.

### Robustness against decoherence/losses

**Figure 4:**
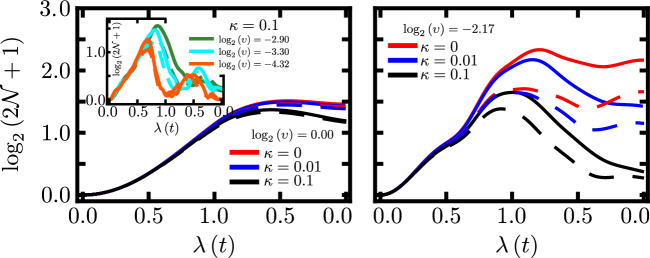
Evidence of robustness against decoherence/losses of the many-body electronic-vibrational entanglement (i.e. quantum coherence) as measured by quantum logarithmic negativity (see text). Results are shown for two representative intermediate up-down pulse durations (i.e. different *v* in left and right panels). Results shown for $N=5$ (dashed lines) and $N=11$ (solid lines), and various values of decoherence $\kappa$. As decoherence increases, the differences between the curves for different *N* tend to become smaller: this hints at a possible universality in robustness with increasing $\kappa$. Inset: largest $\kappa$ value and different $\upsilon$. We find that increasing temperature (and hence $\langle n\rangle$) shows broadly similar tendencies to the results shown for increasing $\kappa$. Figures created by the authors using Mathematica version 13 https://www.wolfram.com/mathematica/ and also Microsoft Powerpoint version 16 https://www.microsoft.com/en-us/microsoft-365.

The presence of decoherence/losses to and from the environment, does not change our main conclusions, as illustrated in Fig. 4. Since $S_N$ is no longer a good entanglement measure in an open system, we use the closely related quantity quantum negativity^71^ ${\mathscr {N}}\left( \rho \right) =\frac{1}{2}(\left\| \rho ^{\Gamma _q} \right\| _{1}-1)$ where $\rho ^{\Gamma _q}$ is the partial transpose of $\rho$ with respect to the matter subsystem, and $\left\| A \right\| _{1}\equiv \textrm{tr} \left\{ \sqrt{A^{\dagger }A} \right\}$ is the trace norm. It gives essentially the same results as $S_N$ for small decoherence since both measures represent essentially the same information^50^. The electronic-vibrational density matrix $\rho (t)$ evolves as^72^:

7 $$\begin{aligned} {{\dot{\rho }}} = - i \left[ H,\rho \right] +2\kappa \left( {\bar{n}}+1 \right) {\mathscr {L}} \left( \rho ;a \right) +2\kappa {\bar{n}} {\mathscr {L}} \left( \rho ;a^{\dagger } \right) , \end{aligned}$$

where $\kappa$ is the damping rate and ${\bar{n}}$ is the thermal mean photon number. The Lindblad superoperator ${\mathscr {L}}$ is defined as $A \rho A^{\dagger }-\frac{1}{2} \left\{ A^{\dagger }A,\rho \right\}$ ($\left\{ \bullet ,\bullet \right\}$ is the anti-commutator). Not only do our main results survive well with increasing decoherence $\kappa$, the strength and robustness of the many-body coherence both increase with *N*. (For very large *N*, other system-level decoherence mechanisms may set in). We clarify that in this analysis, we have neglected the energy level fluctuations. A more realistic approach for photosynthetic systems would include (see, for example, Ref.^73^). We have also simulated different temperatures by varying the average number of quantized vibrations ${\bar{n}}$, choosing values typical of the low temperatures in most experimental realizations, and including a thermal distribution in the initial density matrix^58^. Again we find that our main conclusions are qualitatively unchanged.

### Further observations

In addition to the specific results shown above, it is worth noting the following general points:

1. Our focus in this paper is on the question of collective coherence in any setting where there is some pulsed perturbation of the system, in order to explore better the idea of how excitations (e.g. excitons) may ‘ride a wave’ of coherence. However the external profile ${\vec {E}}(t)$ and hence $\lambda (t)$ can be general. It need not be a pulse. Also the application of Eq. (1) could be to transport experiments as well as optical experiments, or any combination of these.
2. There are two ways in which Eq. (1) can be applied: (i) We assume that incident light has already created excitons in our system, hence the initial ground state in Eq. (1) is one in which each of the *N* nanostructure components is in the lower excited state, i.e. $\left| J,J_z=-N/2 \right\rangle$. An initial excited state in Eq. (1) is one in which one or more of the *N* nanostructure components is in the upper excited state, i.e. $\left| J,J_z>-N/2 \right\rangle$. Our model then calculates what additional many-body coherence is generated by a pulse. (ii) We can alternatively assume that the system starts in a true ground state with no excitations. The same analysis follows. It is case (i) that would be relevant for the particular setting of LHC-II, since it is the energy separation of each dimer’s two hybridized excitonic states $|Y\rangle$ and $|X\rangle$ that is quasi-resonant with a vibrational mode.
3. The pulse $\lambda (t)$ is a consequence of the internal polarization ${\vec {P}}(t)$ due to an external pulse of light ${\vec {E}}(t)$. It is well known that Maxwell’s equations give a nonlinear, exact equation that relates ${\vec {P}}(t)$ to ${\vec {E}}(t)$, and as a result of the mathematical form (see above) a pulse in ${\vec {E}}(t)$ will generate a pulse in ${\vec {P}}(t)$. This justifies the appearance of the pulse in our time-dependent Hamiltonian.
4. The effect of finite temperature is treated when discussing the losses/decoherence, by including finite numbers of vibrational quanta consistent with temperature distributions. We find that these do not change our main conclusions for reasonably low temperatures. Even for higher temperatures, the effects that we discuss have not disappeared.
5. Several papers (e.g. Ref.^2,3,7^) make the point that a deeper understanding of the temporal quantum evolution in systems of general size *N* – as this study tries to offer – may shed light on the early-time kinetics in real-world open systems, since this timescale is too short to couple in many of the complex degrees of freedom that will naturally exist in a hot, wet environment.

## Summary and perspectives

We have demonstrated that pulse-driven perturbations give rise to novel dynamical phenomena linked to many-body ($N\ge 3$) entanglement, specifically quantum coherence. These phenomena can be harnessed for energy harvesting, manipulation, and the design of quantum information devices, all without the necessity of strong electronic-vibrational coupling. By contrast, existing theoretical investigations tend to concentrate on scenarios where $N\rightarrow 1$ and/or use perturbation theory and/or average over memory effects, and hence have not yet uncovered these intriguing new physical phenomena reported here – despite potentially offering a more chemically detailed picture of the system itself.

### Supplementary Information

Supplementary Information.

## Data Availability

The data generated during the current study are available from the corresponding author on reasonable request. All the data and results during the current study are generated by the mathematical model and code.

## Code availability

The codes for this study are available from the corresponding author on reasonable request.

## References

[1] Nishida J (2022). Ultrafast infrared nano-imaging of far-from-equilibrium carrier and vibrational dynamics. Nat. Commun..

[2] Cao J (2020). Quantum biology revisited. Sci. Adv..

[3] Lambert N (2013). Quantum biology. Nat. Phys..

[4] Scholes GD (2017). Using coherence to enhance function in chemical and biophysical systems. Nature.

[5] O’Reilly EJ, Olaya-Castro A (2014). Non-classicality of the molecular vibrations assisting exciton energy transfer at room temperature. Nat. Commun..

[6] Plenio MB, Almeida J, Huelga SF (2013). Origin of long-lived oscillations in $$2d$$-spectra of a quantum vibronic model: electronic versus vibrational coherence. J. Chem. Phys..

[7] Zigmantas D, Polívka T, Persson P, Sundström V (2022). Ultrafast laser spectroscopy uncovers mechanisms of light energy conversion in photosynthesis and sustainable energy materials. Chem. Phys. Rev..

[8] Olaya-Castro A, Lee CF, Olsen FF, Johnson NF (2008). Efficiency of energy transfer in a light-harvesting system under quantum coherence. Phys. Rev. B.

[9] Thilagam A (2015). Natural light harvesting systems: Unraveling the quantum puzzles. J. Math. Chem..

[10] Fujihashi Y, Fleming GR, Ishizaki A (2015). Impact of environmentally induced fluctuations on quantum mechanically mixed electronic and vibrational pigment states in photosynthetic energy transfer and 2d electronic spectra. J. Chem. Phys..

[11] Chin AW (2012). The role of non-equilibrium vibrational structures in electronic coherence and recoherence in pigment-protein complexes. Nat. Phys..

[12] Scholes GD, Fleming GR, Olaya-Castro A, van Grondelle R (2011). Lessons from nature about solar light harvesting. Nat. Chem..

[13] Engel GS (2007). Evidence for wavelike energy transfer through quantum coherence in photosynthetic systems. Nature.

[14] Collini E (2010). Coherently wired light-harvesting in photosynthetic marine algae at ambient temperature. Nature.

[15] Rozzi CA (2013). Quantum coherence controls the charge separation in a prototypical artificial light-harvesting system. Nat. Commun..

[16] Tiwari V, Peters WK, Jonas DM (2012). Electronic resonance with anticorrelated pigment vibrations drives photosynthetic energy transfer outside the adiabatic framework. Proc. Nat. Acad. Sci. USA.

[17] Schlau-Cohen GS (2012). Elucidation of the timescales and origins of quantum electronic coherence in lhcii. Nat. Chem..

[18] Brédas JL, Sargent EH, Scholes GD (2016). Photovoltaic concepts inspired by coherence effects in photosynthetic systems. Nat. Mater..

[19] Scholes GD, Fleming GR (2000). On the mechanism of light-harvesting in photosynthetic purple bacteria: B800 to b850 energy transfer. J. Phys. Chem. B.

[20] Hainer F (2021). Vibrational coherence spectroscopy identifies ultrafast branching in an iron(ii) sensitizer. J. Phys. Chem. Lett..

[21] Reutzel M, Li A, Petek H (2019). Coherent two-dimensional multiphoton photoelectron spectroscopy of metal surfaces. Phys. Rev. X.

[22] Bittner ER, Silva C (2014). Noise-induced quantum coherence drives photo-carrier generation dynamics at polymeric semiconductor heterojunctions. Nat. Commun..

[23] Liedy F (2020). Vibrational coherences in manganese single-molecule magnets after ultrafast photoexcitation. Nat. Chem..

[24] Rogers M (2023). A hybrid magneto-optic capacitive memory with picosecond writing time. Adv. Funct. Mater..

[25] Canton SE (2023). Ultrafast jahn-teller photoswitching in cobalt single-ion magnets. Adv. Sci..

[26] Paulus BC, Adelman SL, Jamula L, McCusker J (2020). Leveraging excited-state coherence for synthetic control of ultrafast dynamics. Nature.

[27] Coccia E, Corni S (2019). Role of coherence in the plasmonic control of molecular absorption. J. Chem. Phys..

[28] Gaynor JD, Sandwisch J, Khalil M (2019). Vibronic coherence evolution in multidimensional ultrafast photochemical processes. Nat. Commun..

[29] Wang H, Valkunas L, Cao T, Whittaker-Brooks L, Fleming G (2016). Coulomb screening and coherent phonon in methylammonium lead iodide perovskites. J. Phys. Chem. Lett..

[30] Liu C (2016). Engineering nanometre-scale coherence in soft matter. Nat. Chem..

[31] Bian Q (2020). Vibronic coherence contributes to photocurrent generation in organic semiconductor heterojunction diodes. Nat. Commun..

[32] Dubin F (2006). Macroscopic coherence of a single exciton state in an organic quantum wire. Nat. Phys..

[33] Collini E, Scholes GD (2009). Coherent intrachain energy migration in a conjugated polymer at room temperature. Science.

[34] Cassette E, Pensack RD, Mahler B, Scholes GD (2015). Room-temperature exciton coherence and dephasing in two-dimensional nanostructures. Nat. Commun..

[35] Scholes GD, Rumbles G (2006). Excitons in nanoscale systems. Nat. Mater..

[36] Tanimura Y (2020). Numerically “exact” approach to open quantum dynamics: The hierarchical equations of motion (HEOM). J. Chem. Phys..

[37] Lambert N, Ahmed S, Cirio M, Nori F (2019). Modelling the ultra-strongly coupled spin-boson model with unphysical modes. Nat. Commun..

[38] Egger R, Mak CH (1994). Low-temperature dynamical simulation of spin-boson systems. Phys. Rev. B.

[39] Cao J, Ungar LW, Voth GA (1996). A novel method for simulating quantum dissipative systems. J. Chem. Phys..

[40] Makri N (1995). Numerical path integral techniques for long time dynamics of quantum dissipative systems. J. Math. Phys..

[41] Makri N, Makarov DE (1995). Tensor propagator for iterative quantum time evolution of reduced density matrices. I. Theory. J. Chem. Phys..

[42] Zhao Y (2023). The hierarchy of Davydov’s Ansätze: From guesswork to numerically “exact” many-body wave functions. J. Chem. Phys..

[43] Liu Z (2004). Crystal structure of spinach major light-harvesting complex at 2.72a resolution. Nature.

[44] Drop, B. *et al.* Light-harvesting complex II (LHCII) and its supramolecular organization in chlamydomonas reinhardtii. *Biochimica et Biophysica Acta (BBA) - Bioenergetics* **1837**, 63–72, 10.1016/j.bbabio.2013.07.012 (2014). 10.1016/j.bbabio.2013.07.012 23933017

[45] Lambrev PH (2007). Importance of trimer-trimer interactions for the native state of the plant light-harvesting complex II. Biochimica et Biophysica Acta (BBA) - Bioenergetics.

[46] Duan H-G (2017). Nature does not rely on long-lived electronic quantum coherence for photosynthetic energy transfer. Proc. Natl. Acad. Sci..

[47] Kenrow JA, El Sayed K, Stanton CJ (1997). Interaction induced electron self-interference in a semiconductor: The phonon staircase effect. Phys. Rev. Lett..

[48] Li C (2017). Nonlinear Optics: Principles and Applications.

[49] Schröter M (2015). Dissipative Exciton Dynamics in Light-Harvesting Complexes.

[50] Gómez-Ruiz FJ, Acevedo OL, Rodríguez FJ, Quiroga L, Johnson NF (2018). Pulsed generation of quantum coherences and non-classicality in light-matter systems. Front. Phys..

[51] Rodríguez FJ (2008). Control of non-markovian effects in the dynamics of polaritons in semiconductor microcavities. Phys. Rev. B.

[52] Acevedo O, Quiroga L, Rodríguez F, Johnson N (2014). New dynamical scaling universality for quantum networks across adiabatic quantum phase transitions. Phys. Rev. Lett..

[53] Lee CF, Johnson NF (2007). Spin-glasses in optical cavity. EPL (Europhys. Lett.).

[54] Jarrett TC, Lee CF, Johnson NF (2006). Optically controlled spin glasses in multiqubit cavity systems. Phys. Rev. B.

[55] Lee CF, Johnson NF (2004). First-order superradiant phase transitions in a multiqubit cavity system. Phys. Rev. Lett..

[56] Jarrett TC, Olaya-Castro A, Johnson NF (2007). Optical signatures of quantum phase transitions in a light-matter system. Europhys. Lett. (EPL).

[57] Acevedo OL, Quiroga L, Rodríguez FJ, Johnson NF (2015). Robust quantum correlations in out-of-equilibrium matter–light systems. New J. Phys..

[58] Acevedo OL, Quiroga L, Rodríguez FJ, Johnson NF (2015). Large dynamic light-matter entanglement from driving neither too fast nor too slow. Phys. Rev. A.

[59] Gómez-Ruiz F, Acevedo O, Quiroga L, Rodríguez F, Johnson N (2016). Quantum hysteresis in coupled light–matter systems. Entropy.

[60] Gómez-Ruiz FJ (2017). Dynamics of entanglement and the schmidt gap in a driven light–matter system. J. Phys. B: At. Mol. Opt. Phys..

[61] Méndez-Córdoba FPM (2020). Rényi entropy singularities as signatures of topological criticality in coupled photon-fermion systems. Phys. Rev. Res..

[62] Méndez-Córdoba FPM, Rodríguez FJ, Tejedor C, Quiroga L (2023). From edge to bulk: Cavity-induced displacement of topological nonlocal qubits. Phys. Rev. B.

[63] Tarantelli F, Vicari E (2022). Out-of-equilibrium dynamics arising from slow round-trip variations of hamiltonian parameters across quantum and classical critical points. Phys. Rev. B.

[64] De Franco F, Vicari E (2023). Out-of-equilibrium finite-size scaling in generalized kibble-zurek protocols crossing quantum phase transitions in the presence of symmetry-breaking perturbations. Phys. Rev. B.

[65] Rey AM, Jiang L, Lukin MD (2007). Quantum-limited measurements of atomic scattering properties. Phys. Rev. A.

[66] Hardal, A. U. C. & Özgür E. Müstecaplıoğlu. Superradiant quantum heat engine. *Sci. Rep.*, 10.1038/srep12953 (2015). 10.1038/srep12953 PMC4531314 26260797

[67] Niedenzu W, Gelbwaser-Klimovsky D, Kurizki G (2015). Performance limits of multilevel and multipartite quantum heat machines. Phys. Rev. E.

[68] Viehmann O, von Delft J, Marquardt F (2011). Superradiant phase transitions and the standard description of circuit qed. Phys. Rev. Lett..

[69] Reslen J, Quiroga L, Johnson NF (2005). Direct equivalence between quantum phase transition phenomena in radiation-matter and magnetic systems: Scaling of entanglement. Europhys. Lett. (EPL).

[70] Scully MO, Zubairy MS (1997). Quantum Optics.

[71] Vidal G, Werner RF (2002). Computable measure of entanglement. Phys. Rev. A.

[72] Breuer H, Petruccione F (2002). The Theory of Open Quantum Systems.

[73] Shim S, Rebentrost P, Valleau S, Aspuru-Guzik A (2012). Atomistic study of the long-lived quantum coherences in the fenna-matthews-olson complex. Biophys. J ..

