# Supplementary material for: Energy transfer in N-component nanosystems enhanced by pulse-driven vibronic many-body entanglement
Source: Sci Rep. 2023 Nov 15;13:19790. doi: 10.1038/s41598-023-46256-z (PMC10651905; doi:10.1038/s41598-023-46256-z)
Supplement: Supplementary file 1 — Supplementary Information. [file 41598_2023_46256_MOESM1_ESM.pdf]

Supplementary Material for 'Energy transfer in N-component nanosystems enhanced by pulse-driven vibronic many-body entanglement'

Fernando J. Gómez-Ruiz,[1] Oscar L. Acevedo,[2] Ferney J. Rodríguez,[3] Luis Quiroga,[3] and Neil F. Johnson[4,*]

Here we provide additional details behind the theory presented in the main paper.

I. TEMPORAL COUPLING $\lambda(t)$ FROM DRIVING FIELD

Here we discuss from a quantum mechanical starting point, the fact that memory effects can arise in the exciton-vibration (XV) dynamics due to the interaction with a (controllable) exterior field – and hence justify the use of a time-dependent $\lambda(t)$ in Eq. (1) of the paper. For quantum systems embedded in complex environments, where extra degrees of freedom modulate the interaction between the quantum system of interest and a large reservoir, effective non-Markovian behaviors in the quantum system dynamics arise even though the reservoir itself can be described within a Markovian approximation. In our model, the memory effects are due to the parametric pulsed coupling between the exciton and the vibration modes which is represented by the time-dependent XV coupling. Consequently, although it is true that the phase imprinted by the excitation laser is lost during the first steps of electron-exciton relaxation from the high energy sector to the XV region, this is not a sufficient reason to exclude any coherent-like behavior in the relaxing XV dynamics. Indeed it can be shown that for a wide class of phase-mixed states of the pump modes, results for the signal population can be obtained that are identical to those for a coherent population of those modes. In order to clarify this critical point, we now show that our basic premise is justified for a variety of reasons. According to the extensive literature concerning light-matter Hamiltonians, in the classical limit the system can be considered as equivalent to two coupled harmonic oscillators. This information is enough to gain analytical insight into the solution of the resulting quadratic system. The driven system in this limit is described by two coupled harmonic oscillators with a time-dependent coupling frequency. Consequently for this purpose, we will consider a simplified model for the parametric process that contains just 3 boson modes (for the sake of simplicity we describe now the N dimer subsystem in the low excitation limit as an effective boson b mode), as described by the Hamiltonian:

$$\hat{H} = \omega_a \hat{a}^\dagger \hat{a} + \chi \left(\hat{a}^\dagger \hat{a}\right)^2 + \omega_b \hat{b}^\dagger \hat{b} + \omega_c \hat{c}^\dagger \hat{c} + g\left(\hat{a}^\dagger \hat{b}^\dagger \hat{c}^2 + \hat{a}\hat{b}\hat{c}^{\dagger 2}\right) \tag{1}$$

where the operators $\hat{a}$, $\hat{b}$ and $\hat{c}$ correspond to the vibration, exciton and high energy controllable exciton modes, where we allow for anharmonic terms of strength χ for the vibration mode. We now consider the effect of the pump state on the dynamics of this simple, but representative, model. In particular, we consider the excitation of high energy electron states, which indirectly feeds (through relaxation process) an effective pump reservoir which follows the applied radiation pulse shape. We assume in Eq. (1) that $\left(\hat{a}^\dagger \hat{b}^\dagger \hat{c}^2 + \hat{a}\hat{b}\hat{c}^{\dagger 2}\right) = h(t)(\hat{a}^\dagger \hat{b}^\dagger + \hat{a}\hat{b})$ where $h(t)$ represents the applied pulse shape. It is usually argued that the expectation value $\langle \hat{c}^{\dagger 2}\rangle$ ($\langle \hat{c}^2\rangle$) is different from zero only if the high energy reservoir states have coherent populations. Since the laser pulse excites electrons at a higher energy, the excess energy might be expected to relax into the exciton region giving rise to a coherent interaction. However this is not necessarily the case: after non-resonant excitation, the phase imprinted by the excitation laser is generally lost. The appearance of a well-defined phase is often regarded as the true characteristic feature of a coherent state. However, a careful analysis of unitary dynamics from mixed states, such as those produced by incoherent relaxation processes, shows that coherent-like behaviors can often be obtained. In order to justify this last claim we compute the time evolution of XV observables under two kind of pump initial states: (i) A pure initial state like $|\,\Psi\rangle = |\,0_a\rangle\,|\,0_b\rangle\,|\,\alpha_c\rangle$, denoting the vacuum state for both XV modes, and a pump coherent state. (ii) A statistical mixed state with no phase information at all, given by a density matrix $\hat{\rho}_P = \int_0^{2\pi} d\theta P(\theta)\hat{\Pi}_p(\theta)\,|\,\Psi\rangle\langle\Psi\,|\,\hat{\Pi}_p^{-1}(\theta)$, where $\hat{\Pi}_p(\theta) = e^{i\hat{N}\theta}$, with $\hat{N} = \hat{a}^\dagger\hat{a} + \hat{b}^\dagger\hat{b} + \hat{c}^\dagger\hat{c}$, denotes a phase smearing operator, given the fact that it takes a pump coherent state $|\,\alpha_c\rangle$ to a different phase coherent state $|\,e^{i\theta}\alpha_c\rangle$, leaving the XV modes in the vacuum state. The function $P(\theta)$ fixes the pump phase smearing effect with $P(\theta) \geq 0$ and $\int_0^{2\pi} d\theta P(\theta) = 1$. Since $\left[\hat{H}, \hat{N}\right] = 0$, it follows that the time-evolution operator $\hat{U}(t) = e^{-i\hat{H}t}$ commutes with the phase smearing operator $\hat{\Pi}_p(\theta)$. It is now an easy task to obtain for any XV observable like

* neiljohnson@gwu.edu

$\hat{a}^{\dagger k}\hat{a}^l$, the time-evolution as

$$\begin{aligned}\langle \hat{a}^{\dagger k}\hat{a}^l\rangle_P =& Tr\{\hat{a}^{\dagger k}\hat{a}^l\hat{\rho}_P(t)\} \\ =& \int_0^{2\pi} d\theta P(\theta) Tr\{\hat{U}^{-1}(t)\hat{\Pi}_p^{-1}(\theta)\hat{a}^{\dagger k}\hat{a}^l\hat{\Pi}_p(\theta)\hat{U}(t)|\Psi\rangle\langle\Psi|\}\,.\end{aligned} \tag{2}$$

Since $\hat{\Pi}_p^{-1}(\theta)\hat{a}^{\dagger k}\hat{\Pi}_p(\theta) = e^{-ik\theta}\hat{a}^{\dagger k}$ and $\hat{\Pi}_p^{-1}(\theta)\hat{a}^l\hat{\Pi}_p(\theta) = e^{il\theta}\hat{a}^l$ it follows that

$$\langle \hat{a}^{\dagger k}\hat{a}^l\rangle_P = \langle \hat{a}^{\dagger k}\hat{a}^l\rangle_0 \int_0^{2\pi} d\theta P(\theta) e^{-i(k-l)\theta} \tag{3}$$

where $\langle \hat{a}^{\dagger k}\hat{a}^l\rangle_0$ corresponds to the initial state with the pump in a coherent state. From Eq. (3) it is evident that the population dynamics of the vibrational subsystem ($k = l = 1$) is fully insensitive to this class of phase smearing in the pump state, $\langle \hat{a}^\dagger\hat{a}\rangle_P = \langle \hat{a}^\dagger\hat{a}\rangle_0$, as well as other vibrational correlations as long as the pump phase smearing probability $P(\theta)$ remains practically constant. The main physical ingredients of a general, complex XV light-matter system can therefore be captured by this simple 3-mode Hamiltonian. Therefore we can conclude that for a wide class of coherent pump-plus-relaxation process conditions, our main results on the non-Markovian evolution of the vibrational population are indeed meaningful. Hence the replacement of $\hat{c}$-pump operators by complex numbers – which consequently yields a time-dependent XV coupling strength $\lambda(t)$ – is justified. Also, the range of validity of our assumption is the same as the usual one for the parametric approximation which requires a highly populated coherent state, $|\alpha_c| \gg 1$, and short times, $gt \ll 1$. These conditions are precisely identical to those under which we show our model fits with previous studies of XV coherence generation: high excitation and a rapid relaxation dynamics. Therefore, there is indeed a formal justification for reducing the last terms in Eq. (1) to $gh(t)(\hat{a}^\dagger\hat{b}^\dagger + \hat{a}\hat{b})$ where $h(t)$ represents the applied pulse shape – hence justifying the time-dependent interaction $\lambda(t) \sim gh(t)$ in Eq. 1 of the main paper.

Next we discuss the parallel statement in the main paper, that any incident electromagnetic (light) field $\vec{E}$ generates an internal polarization field $\vec{P}$ within the material, given exactly by Maxwell's Equations. Following Ref. 37, the equation describing the time-domain behavior in a general, anisotropic and nonlinear medium subject to a general time and position-dependent light field $\vec{E}$ is given by:

$$\nabla\times\nabla\times\vec{E} + \mu_0\sigma\frac{\partial\vec{E}}{\partial t} + \mu_0\frac{\partial^2\vec{\vec{\epsilon}}\cdot\vec{E}}{\partial^2 t} = -\mu_0\frac{\partial^2\vec{P}}{\partial^2 t}$$

which is Eq. (2.1.17) from Ref. 37 in which the standard symbols have their well-known meaning from electromagnetic theory (e.g. $\vec{\vec{\epsilon}}$ is a complex second-order tensor). Following Ref. 37, if the medium is lossless then $\sigma = 0$ and so this equation can be rewritten as:

$$[\nabla\times(\nabla\times) + \frac{1}{\epsilon_0 c^2}\frac{\partial^2}{\partial t^2}\vec{\vec{\epsilon}}\cdot]\vec{E} = -\frac{1}{\epsilon_0 c^2}\frac{\partial^2\vec{P}}{\partial^2 t}$$

which is Eq. (2.1.18) from Ref. 37, and also Eq. (2.130) of Ref. 38. Though nonlinear and anisotropic in general, the presence of $\partial^2/\partial t^2$ terms for $\vec{E}$ and $\vec{P}$ in both equations means that a pulse in $\vec{E}$ will generate a similar pulse in $\vec{P}$, and hence a pulse in the internal electric field dynamics coupling the electronic and vibrational systems (i.e. a pulse in $\lambda(t)$).

II. MULTI-COMPONENT RESONANCE

Our focus is on near resonant conditions since these are the most favorable for generating large coherences. We only consider one such resonance for simplicity, however this can be generalized by matching up different excitation energies ϵ', ϵ'', etc. to the nearest vibrational energies ω', ω'' etc. and then solving Eq. 1 in the same way for each subset (ϵ', ω') etc. For example, if the N components are partitioned into n subpopulations, where each subpopulation has the same resonant energy and vibrational mode but where these values differ between subpopulations, the total Hamiltonian will approximately decouple into $H^{(1)} \oplus H^{(2)} \oplus H^{(3)} \cdots \oplus H^{(N)}$. Any residual coupling between these subpopulations can then be treated as noise.

III. CALCULATION DETAILS

For each time t starting at $t = 0$, we obtain numerically the instantaneous state $|\psi(t)\rangle$. The accuracy of our numerical solutions was checked by extending the expansion basis beyond the point of convergence. We obtain the full instantaneous state $|\psi(t)\rangle$ by numerically solving the time-dependent Schrödinger equation. Our numerical solution profits from the fact that the operator $\hat{\mathbf{J}}^2 = \sum_\alpha \hat{J}_\alpha^2$ is a constant of motion with eigenvalue $J(J+1)$, and that the parity operator $\hat{\mathcal{P}} = \exp\left(\imath\pi\left[\hat{a}^\dagger\hat{a} + J_z + J\right]\right)$ is also conserved and commutes with $\hat{\mathbf{J}}^2$. Since we are seeking results that have general validity, we avoid making the rotating-wave approximation that is commonly used to solve the static version of the model and which makes it Bethe ansatz integrable. In the thermodynamic limit $N \to \infty$, the static-DM exhibits a second-order quantum phase transition (QPT) at $\lambda_c = \sqrt{\epsilon\omega}/2$ with order parameter $\hat{a}^\dagger\hat{a}/J$, separating the normal phase at $\lambda_c < \sqrt{\epsilon\omega}/2$ from the superradiant phase in which there is a finite value of the macroscopic order parameter, e.g. finite boson expectation number. The general structure of the state $|\psi(t)\rangle$ at any time t is given by

$$|\psi(t)\rangle = \sum_{m_z=-N/2}^{N/2}\sum_{n=0}^{\chi} C_{n,m_z}(t)\,|m_z, n\rangle\,. \tag{4}$$

Here χ is the truncation parameter of the size of the bosonic Fock space, whose value we choose to be large enough to ensure that the numerical results converge.

The basis states $|m_z, n\rangle = |m_z\rangle \otimes |n\rangle$ are defined such that $|m_z\rangle$ is an eigenvector of J_z in the subspace of even parity with eigenvalue m_z, and $|n\rangle$ is a bosonic Fock state with occupation n. The initial state of the dynamics at $t = 0$, with negligible electronic-vibrational coupling $\lambda(t) = 0$, is the non-interacting ground state $|\psi(0)\rangle = \bigotimes_{i=1}^{N} |\downarrow\rangle \otimes |n = 0\rangle = \left|-\frac{N}{2}, 0\right\rangle$. All two-level systems are polarized in the state with $\langle\sigma_z\rangle = -1$, and the vibrational field is in the Fock state of zero excitations. Since the total angular momentum and parity are conserved quantities, we can without loss of generality restrict our study to the maximum angular momentum sector $J = N/2$ and $\mathcal{P} = 1$. We obtain the time-evolution of the system in response to an up-down pulse in $\lambda(t)$, which is chosen so that the system dynamically crosses the QPT on both the up and down portion of the $\lambda(t)$ pulse cycle. For simplicity, we consider $\lambda(t)$ to rise and fall linearly in time t during the pulse, i.e. it has the form vt, and thus establishes a triangular ramping of the electronic-vibrational interaction.

Given a subsystem A, the von Neumann entropy:

$$S_N = -\mathrm{tr}\left\{\rho_A \log\left(\rho_A\right)\right\}\ ,\quad \rho_A = \mathrm{tr}_B\left\{|\psi\rangle\langle\psi|\right\} \qquad (5)$$

where B is the complementary subsystem and the total system is in a total state $|\psi\rangle$ that is pure. When the total system is in such a pure state, the entropy of subsystem A is equal to the entropy of its complementary subsystem B, and this quantity S_N is a measure of the entanglement between both subsystems. The natural choice in our system for such a bipartition is where one subsystem is the vibrational mode and the other subsystem is the molecular excitonic subsystem. Since this a closed system (i.e. a pure global quantum state with an unitary evolution), the increase of S_N in each subsystem is synonymous with an interchange of information between the vibrations and molecular components during the cycle, hence providing a more direct thermodynamical interpretation for the memory effects of the cycle.

Our calculation of decoherence is consistent with state-of-the-art calculations of decoherence in the literature. Like these others, of course, it is not perfect in that decoherence should, in principle, include all non-resonant dimers etc. as well as extending the possible admixture of states to those outside the Dicke manifold and hence all possible sectors of the angular momentum. This would render our calculation of decoherence, which already takes of order weeks to complete on a large computer cluster, impossible: hence we instead follow the sensible route of including all states within the Dicke manifold as well as cavity losses in the bosonic vibrational system.
